# Layer-specific femorotibial cartilage T2 relaxation time in knees with and without early knee osteoarthritis: Data from the Osteoarthritis Initiative (OAI)

**DOI:** 10.1038/srep34202

**Published:** 2016-09-27

**Authors:** W. Wirth, S. Maschek, F. W. Roemer, F. Eckstein

**Affiliations:** 1 Institute of Anatomy, Paracelsus Medical University Salzburg & Nuremberg, Salzburg, Austria; 2 Chondrometrics GmbH, Ainring, Germany; 3 Department of Radiology, University of Erlangen-Nuremberg, Erlangen, Germany; 4 Quantitative Imaging Center (QIC), Department of Radiology, Boston University School of Medicine, Boston, MA, USA

## Abstract

Magnetic resonance imaging (MRI)-based spin-spin relaxation time (T2) mapping has been shown to be associated with cartilage matrix composition (hydration, collagen content & orientation). To determine the impact of early radiographic knee osteoarthritis (ROA) and ROA risk factors on femorotibial cartilage composition, we studied baseline values and one-year change in superficial and deep cartilage T2 layers in 60 subjects (age 60.6 ± 9.6 y; BMI 27.8 ± 4.8) with definite osteophytes in one knee (earlyROA, n = 32) and with ROA risk factors in the contralateral knee (riskROA, n = 28), and 89 healthy subjects (age 55.0 ± 7.5 y; BMI 24.4 ± 3.1) without signs or risk factors of ROA. Baseline T2 did not differ significantly between earlyROA and riskROA knees in the superficial (48.0 ± 3.5 ms vs. 48.1 ± 3.1 ms) or the deep layer (37.3 ± 2.5 ms vs. 37.3 ± 1.8 ms). However, healthy knees showed significantly lower superficial layer T2 (45.4 ± 2.3 ms) than earlyROA or riskROA knees (p ≤ 0.001) and significantly lower deep layer T2 (35.8 ± 1.8 ms) than riskROA knees (p = 0.006). Significant longitudinal change in T2 (superficial: 0.5 ± 1.4 ms; deep: 0.8 ± 1.3 ms) was only detected in healthy knees. These results do not suggest an association of early ROA (osteophytes) with cartilage composition, as assessed by T2 mapping, whereas cartilage composition was observed to differ between knees with and without ROA risk factors.

Articular cartilage spin-spin (transverse) relaxation time (T2) is known to be associated with cartilage composition (hydration, collagen integrity and orientation)^1^,^2^,^3^ and has been shown to correlate with histological grading^4^,^5^ and cartilage mechanical properties^2^,^6^. T2 has thus gained interest as an imaging biomarker for detecting and monitoring “early” stages of osteoarthritis (OA)^2^,^3^,^7^, a stage at which therapeutic intervention is potentially more successful than at more advanced stages of the disease. Several studies have reported T2 to differentiate between subjects with and without radiographic OA (ROA) in femorotibial^8^,^9^, patellar^10^, acetabular^11^, and gleno-humeral cartilage^12^. Yet, other studies were unable to confirm differences in cartilage T2 between subjects with and without ROA^13^, or found T2 to not differentiate between different stages of OA^8^. Further, knee cartilage T2 was reported to be longer and more heterogeneous in subjects at risk of developing OA than in healthy reference subjects, despite similar prevalence of cartilage, bone marrow or meniscus lesions^14^.

However, few studies have differentially analyzed laminar (superficial and deep zone) cartilage T2 in context of ROA status, although it has been recognized that (a) superficial cartilage displays significantly longer T2 than deep zone cartilage^2^,^15^; (b) spatial assessment of knee cartilage T2 using laminar and texture analysis may improve discrimination of cartilage matrix abnormalities in OA^16^, and (c) superficial zone cartilage was more sensitive to the presence of semi-quantitatively graded cartilage lesions than deep layer cartilage, and thus potentially more sensitive in detecting compositional differences of the cartilage in the early stages of knee OA^17^.

The purpose of the current study therefore was to investigate whether superficial and deep zone femorotibial cartilage T2 times differ cross-sectionally between knees with and without early ROA, and/or between knees with and without risk factors of OA. Secondly, we studied whether longitudinal change in cartilage T2 times over 1-year differ between knees with and without early ROA, and/or between knees with and without risk factors of OA.

## Methods

### Study participants

The participants for this analysis were selected from the Osteoarthritis Initiative cohort (OAI; http://www.oai.ucsf.edu/, clinicaltrials.gov identifier: NCT00080171)^18^. The OAI was approved by the Committee on Human Research, the Institutional Review Board for the University of California, San Francisco (UCSF). All OAI participants provided written informed consent and this study was carried out in accordance with the IRB-approved OAI data user agreement. At baseline, the OAI cohort included 4796 participants aged 45–79 years that were recruited at one of four clinical sites^18^. General exclusion criteria of the OAI were presence of rheumatoid or other inflammatory arthritis, bilateral end-stage knee OA, inability to walk without aids, and MRI contraindications^18^. Of the 4796 participants, the 1390 participants enrolled in the progression cohort had both symptomatic (i.e. pain, aching or stiffness in the past year) and radiographic OA (osteophytes and/or joint space narrowing in fixed-flexion radiographs) in one or both of their knees. The 3284 incidence cohort participants were at risk of developing knee OA, but did not have both symptomatic and radiographic OA at baseline in either knee. The remaining 122 participants of the OAI were selected as “non-exposed”, healthy controls and had no radiographic abnormalities in either knee according to the OAI clinical site readings^18^. These participants also were free of clinical signs of knee OA, and not exposed to risk factors for developing knee OA, such as obesity, knee injury, knee surgery, a family history of TKA in a biological parent or sibling, Heberden’s nodes, or repetitive knee bending during daily activities. For further details on these OA cohorts, please see ref. 18. At each of 5 subsequent annual visits (baseline through 48 month follow-up), the OAI collected clinical data and acquired both 3T MRI of the knees^19^ and bilateral fixed-flexion radiographs.

The primary comparison of knees with and without early ROA was performed in a cohort of OAI participants with unilateral early ROA that has been described in previous publications^20^,^21^, and was selected based on the following criteria:

1. A definite osteophyte in one knee (definite ROA), based on the OAI site readings,
2. no definite or possible osteophyte in the contralateral knee,
3. no radiographic joint space narrowing (JSN) in either knee, according to the OARSI atlas.

This specific choice in OAI participants, in particular exclusion of any radiographic JSN, was made, so that the analysis of cartilage T2 was restricted to the early stages of ROA, i.e. formation of an osteophyte in one knee, whereas the other knee still is free of any sign of radiographic change. According to the site radiographic readings, these specific conditions were fulfilled by 84 of the 4796 OAI participants. All cases were then reviewed by an expert musculoskeletal radiologist (F.R.), who confirmed these specific radiographic selection criteria in 61 of the 84 participants^20^,^21^. In previous analyses of this very same sample of 61 subjects^20^,^21^, we observed the cartilage thickness at baseline in the external medial and lateral femur to be greater in earlyROA knees than in contra-lateral riskROA knees^20^. However, we did not observe a significant longitudinal change in femorotibial cartilage thickness in either the earlyROA (osteophyte) knees or in the contralateral riskROA knees over a one year follow-up period, using the same regions of interest as studied here^21^.

Further, our analysis included participants from the OAI “non-exposed”, healthy reference cohort. Of the 122 healthy reference cohort participants, 92 had follow-up MR images and were confirmed to be free of any radiographic abnormalities by the central radiographic readings^18^ in addition to being free of OA risk factors^18^,^22^. Three participants, who developed early signs of radiographic OA (KLG 1) at the 1 year follow-up, were excluded from the analysis, resulting in 89 healthy reference participants included in the analysis.

### MR Imaging

**Figure 1:**
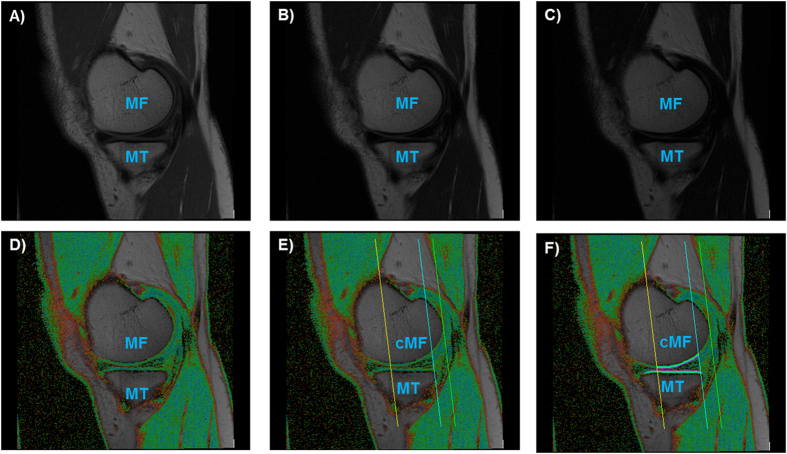
Sagittal multi-echo spin-echo (MESE) images showing the medial tibia (MT) and the medial femur (MF). (**A–C**) MESE images with the shortest (10 ms, **A**), an intermediate (40 ms, **B**), and the longest echo time (70 ms, **C**). (**D**) T2 map of the (medial) femorotibial cartilages, with values demonstrated by color coding. (**E**) T2 map showing the region of interest used to define the central, weight-bearing part of the MF (cMF). (**F**) T2 map showing the segmentation of the MT and the cMF.

Sagittal 3 Tesla multi-echo spin-echo (MESE) MR images were acquired for cartilage T2 analyses in one of the knees of all OAI participants (usually the right knee, Fig. 1)^18^,^19^. The repetition time was 2700 ms, and the echo times were 10, 20, 30, 40, 50, 60, and 70 ms (slice thickness 3.0 mm, in-plane resolution 0.3125 mm). All imaging parameters were kept constant between baseline and follow-up. Baseline MR images for one of the knees were available for 60 of the 61 participants and 1-year follow-up MR images were available for 50 of the 61 participants. In 32 participants, the MESE images (of the right knee) happened to be available for the knee with osteophytes (earlyROA); in 28 participants, the MESE images (of the right knee) happened to be available for the contralateral knee without osteophytes (riskROA). MESE images were available for all 92 healthy reference knees.

### T2 analysis of femorotibial cartilage

Segmentation of the cartilage of the medial and lateral tibia (MT/LT) and the medial and lateral weight-bearing femoral condyles (cMF/cLF) was performed manually in the MESE MR images by experienced readers, by processing all images that displayed tibial and weight-bearing cartilage across the entire knee (Fig. 1)^23^. The tibial cartilage was segmented from its anterior to posterior end, and the femoral cartilage throughout a weight-bearing region of interest, as defined previously^24^. Baseline and follow-up images were displayed simultaneously, in order to ensure a consistent selection of the region of interest analyzed in the longitudinal analysis, but with the readers blinded to acquisition dates in order to exclude bias. Because cartilage T2 is known to display spatial variation with tissue depth^2^,^15^, the segmented cartilages were then computationally divided into the top (superficial) and bottom (deep) 50%, based on the local distance between the segmented cartilage surface and bone interface^23^ (Fig. 1). Cartilage T2 times (in ms) were computed for each voxel by fitting a mono-exponential decay curve to the measured signal intensities using a non-linear method^25^ (1), with the 1^st^ echo (10 ms) excluded to reduce the impact of stimulated echoes^2^. Voxels with R^2^ < 0.66 for the curve fitting were eliminated, to avoid contribution from voxels with low image quality^23^.

### Statistical analysis

Statistical analyses were conducted using IBM SPSS 22 software (IBM Corporation, Armonk, NY). The primary analytic focus was to determine whether baseline T2 values (in ms) in superficial or deep femorotibial cartilage layers averaged over all four femorotibial plates differed between “earlyROA” knees with osteophytes and between “riskROA” knees that were exposed to the same risk factors, but were yet without osteophytes. The co-primary analytic focus was then to compare “earlyROA” and “riskROA” knees with the healthy reference knees not exposed to OA risk factors. In view of the 6 parallel comparison made (3 groups × 2 layers), a p-value of <0.00833 (=0.05/6) was deemed to indicate statistical significance. Crude analyses were performed using unpaired, two-sided t-tests. An ANCOVA was then used to check whether the results were consistent when adjusting for age, sex, and BMI. Cohen D was used as a measure of effect size.

The secondary analytic focus was on whether longitudinal (one year) change in T2 values (in ms) in the superficial or deep femorotibial cartilage layers differed between the three groups and the same statistical approaches were used as for the baseline comparison. To that end, change in T2 was measured in each individual and then averaged across the cohort. A paired, two-sided t-test was used to evaluate within-knee change between baseline and follow-up for each layer and group, with p < 0.05 indicating a significant change over time in a descriptive context. A paired, two-sided t-test was also used to evaluate whether longitudinal change in the superficial layer T2 was stronger than that in deep layer T2. These tests were also performed for the four femorotibial cartilage plates separately, but no further adjustments for multiple parallel statistical testing were made in view of the exploratory nature of these analyses.

## Results

### Demographics and qualitative observations

The 32 earlyROA knees were from participants who were 60.2 ± 10.0 y old, with a BMI of 27.6 ± 4.6 (56% female) and the 28 riskROA knees were from participants who were 61.1 ± 9.4 y old, with a BMI of 28.0 ± 5.0 (50% female, Table 1). The 89 healthy reference participants were 55.0 ± 7.5 y old, with a BMI of 24.4 ± 3.1 (60% female) and hence were significantly younger and less obese (both p ≤ 0.001) than both the riskROA and earlyROA participants. The differences in age and BMI between the riskROA and the earlyROA participants were not statistically significant (p ≥ 0.35). No significant between-group differences were observed for the cartilage thickness or minimum radiographic joint space width at baseline (Table 1). Only one knee from the risk ROA group showed an increase (from KLG 0 to KLG2) over the one-year follow-up period according to the OAI central KLG readings (incident definite medial compartment osteophytes, but no joint space narrowing).

In all groups and cartilage plates, cartilage T2 was longer in the superficial than in the deep cartilage layer (Table 2): In the healthy reference participants, the femorotibial baseline cartilage T2 in the superficial layer was 9.6 ± 2.2 ms longer (95% CI [9.2, 10.1] ms) than in the deep layer and similar differences were observed in the earlyROA (10.7 ± 2.5 ms, 95% CI [9.8, 11.6] ms) and in the riskROA knees (10.8 ± 2.0 ms, 95% CI [10.0, 11.6] ms). Baseline T2 was consistently longer in the weight-bearing femoral cartilage than in the tibial cartilage, with differences of 5–10 ms across cartilage layers (superficial vs. deep), compartments (medial vs. lateral), and groups (Table 2). Baseline cartilage T2 values were, however, similar between the medial and lateral femorotibial compartment, with differences of <3 ms across the different layers, plates and groups (Table 2).

### Baseline between-group T2 analysis

No statistically significant difference in baseline T2 was detected between the 32 earlyROA knees and the 28 riskROA knees from the 60 participants with discordant osteophyte status (Table 2). The mean crude difference between these groups across the femorotibial cartilages was 0.1 ms (95% CI [−1.7, 1.8] ms; p = 0.93) for the superficial layer T2, and 0.0 ms (95% CI [−1.2, 1.1] ms; p = 0.98) for the deep layer T2. The ANCOVA analysis with adjustment for differences in age, sex, and BMI resulted in comparable, non-significant p-values (Table 2).

Overall, the femorotibial T2 values were observed to be shorter in the 92 non-exposed healthy reference cohort knees than in the earlyROA and riskROA knees (Table 2). The mean crude difference between healthy vs riskROA knees was −2.7 ms (95% CI [−3.8, −1.6] ms; p < 0.001) for superficial T2, and −1.5 ms (95% CI [−2.3, −0.7] ms; p < 0.001) for deep layer T2; the difference was greater for superficial (Cohen’s D = 1.04) than for deep cartilage (Cohen’s D = 0.81) and remained statistically significant when adjusting for age, sex, and BMI (Table 2).

The mean crude difference between healthy and earlyROA knees was −2.6 ms (95% CI [−3.7, −1.5] ms; p < 0.001) for superficial layer T2, and −1.5 ms (95% CI [−2.3, −0.7] ms; p < 0.001) for deep layer T2; the difference was again greater for the superficial (Cohen D = 0.96) than for the deep cartilage layers (Cohen D = 0.73). When adjusting for age, sex, and BMI, the difference remained statistically significant for the superficial layer (p < 0.001), but did not reach the adjusted significance level (p < 0.0083) for the deep layer (p = 0.01, Table 2). Table 2 also shows the results for superficial and deep layers in the four femorotibial cartilage plates separately.

### Longitudinal between-group T2 analysis

No statistically significant longitudinal change was noted in either the superficial or deep femorotibial cartilage layers of riskROA (p ≥ 0.27, Table 3) or earlyROA knees (p ≥ 0.41, Table 3). However, a significant increase in T2 between baseline and 1-year follow-up was noted in healthy reference knees across superficial (0.5 ± 1.4 ms; 95% CI [0.2, 0.9] ms; p < 0.001) and deep (0.8 ± 1.3 ms; 95% CI [0.5, 1.1] ms; p < 0.001) femorotibial cartilage (Table 3); the rate of change differed significantly between both layers (p = 0.04). These longitudinal changes in femorotibial cartilage T2 in healthy reference knees were significantly greater than in the riskROA and earlyROA knees (Table 3); however, the difference only remained statistically significant for the deep layer in earlyROA vs. healthy reference knees when adjusting for age, sex, BMI, and multiple comparisons (Table 3). Table 3 also shows the results for superficial and deep layers in the 4 cartilage plates separately.

## Discussion

Using a laminar analysis approach, femorotibial cartilage T2 was not observed to differ significantly between knees with definite early ROA and knees that had risk factors for developing knee OA in cross-sectional analyses, in either superficial or deep femorotibial cartilage layers. It is important to note that, in contrast to previous studies, the knees with and without established ROA had similar risk factors, in that they had an identical contra-lateral knee OA status (no ROA in case of earlyROA knees, and earlyROA for in case of no ROA knees). Preferably, we would have directly compared femorotibial cartilage T2 between the earlyROA and the contralateral riskROA knee of the same person (between-knee, within-person comparison), as previously done for cartilage thickness^20^,^21^. However, this was impossible, because the OAI only acquired MESE images of the right knees in each participant. Yet, comparing right knees with osteophytes (early ROA) and right knees without osteophytes (riskROA) in the above sample with discordant osteophyte status was selected as the “next best” approach, because contra-lateral knee ROA status has been identified as an important predictor of progression of knee OA^26^ and by proceeding as described, knees with and without ROA were both selected based on a similar background of risk factors for developing ROA per inclusion criteria. A potential explanation of the observations made is that T2 changes occur “very early” in the disease process and are irreversible, once present. The lack of differentiation between the earlyROA and riskROA knees may be a result of a ceiling effect, with the cartilage T2 changes having occurred in both earlyROA and riskROA knees already, without progressing any further.

In contrast, femorotibial cartilage T2 differed significantly between knees from the healthy reference cohort when compared to both knees with risk factors for developing OA and established early ROA; this difference remained significant when adjusting for age, sex and BMI. Interestingly, the differences between knees with and without knee OA risk factors were greater for superficial than for deep femorotibial cartilage layers, indicating that superficial T2 maybe more sensitive to variation of cartilage composition with risk factor status of OA. These results extend previous findings of superficial zone cartilage being more sensitive to the presence of semi-quantitatively graded cartilage lesions than deep (bone) layer cartilage^17^.

A limitation of our study is that cartilage lesion scores were not available for the knees studied, but it has been previously reported that OAI knees with risk factors (but without ROA) had similar prevalence of cartilage, bone marrow and meniscus lesions as participants of the OAI healthy reference cohort^14^. Another limitation of this study is the lack of study-specific data on the test-retest precision of cartilage T2 values for the manual segmentation method used in this study. However, previous studies have shown a good test-retest precision for cartilage morphometry analyses using the same quality-controlled manual segmentation method and the same definition for the central, weight-bearing parts of the femur^27^,^28^. In addition, previous studies reported adequate test-retest precision errors for cartilage T2 analyses^2^,^15^,^29^ and the OAI used a continuous quality assurance process to ensure the long-term stability and quality of the MRI acquisitions^30^. Despite discordant ROA status, no significant differences in cartilage T2 times were observed between knees with and contralateral knees without osteophytes (early vs. risk ROA) in the cross-sectional analysis; in contrast, cartilage T2 was significantly lower in knees from the healthy reference cohort than in risk and early ROA knees (with and without osteophytes). The participants in the healthy reference cohort were enrolled based on not being exposed to risk factors of developing OA, whereas the participants with unilateral knee OA were from the OAI incidence or progression cohort and displayed risk factors of incident OA that made them eligible for participating in the OAI. The results therefore indicate that it is the risk factors of incident knee OA that affect the cartilage T2, rather than radiographic status (presence of osteophytes). Previous studies have demonstrated an effect of BMI on cartilage T2^31^,^32^, a known risk factor of incident OA. It is beyond the scope of the current paper to identify specific risk factors that may be responsible for alterations in cartilage T2, but it is important to note that, in the presence of risk factors, presence of osteophytes does not appear to affect cartilage T2. The cross-sectional findings of the current study therefore indicate that the differences in cartilage T2 observed between knees with divergent ROA status reported in previous studies^8^,^9^,^10^,^11^,^12^ may actually be due to differences in risk factor profiles between cohorts rather than due to actual differences in ROA status. This interpretation is supported by a study that identified significant differences in glenohumeral cartilage T2 of subjects with primary OA (which supposedly suffered from common OA risk factors) versus those without OA, but not in those with secondary (post-traumatic) OA versus those without OA^12^. Nevertheless, further studies with larger number of participants should be performed to confirm this hypothesis and to identify the specific set of risk factors responsible for longer cartilage T2.

The results of the longitudinal analysis are somewhat puzzling. Although we have previously shown that the early ROA and non-ROA knees studied here did not display measureable changes in cartilage thickness over one year of follow-up^21^, and although is well known that cartilage T2 increases with age^2^,^33^, we have no convincing explanation why a significant increase was detectable in healthy reference participants over a relatively short (one-year) observation interval, while no change was observed in ROA and non-ROA knees with risk factors of OA. It is, however, unlikely that the increase observed in the healthy reference cohort was caused by the analysis method used in the current study, because the readers were blinded to the acquisition order and dates during the analysis to avoid a potential systematic bias and because random precision errors would not have affected all cartilages in such a consistent manner. Potentially, the “ceiling effect” discussed previously might be responsible for the stable cartilage T2 times observed in the ROA and non-ROA knees. Further, T2 shortening has been observed in some cases when further cartilage degradation occurs. When this is the case, the average T2 of degraded cartilage may actually remain stable, whereas the variability in T2 may continue to increase. Therefore, T2 texture analysis^14^,^34^ of femorotibial cartilage may be used in future studies, to test this hypothesis. Previously, Stahl *et al*. also were unable to demonstrate significant change in T2 over one year in age-matched subjects with and without OA^9^, but the sample (n = 8 vs. 10) was relatively small. Baum *et al*.^35^, in contrast, reported cartilage T2 to significantly increase over 2 years in subjects with and without OA risk factors, but neither presence of risk factors nor the presence of baseline cartilage lesions were significantly associated with the increase in femorotibial cartilage T2. Although one study reported an inverse correlation of longitudinal T2 changes over 2 years versus baseline T2 values and morphological cartilage abnormalities^7^, cartilage lesions and other structural abnormalities on MRI were observed to be similar between healthy reference subjects and those with risk factors of OA (but without ROA)^14^, and thus this observation does not provide a likely explanation for the observed difference in longitudinal T2 change between healthy reference knees and knees with risk factors of knee OA. Hence, further studies are needed to elucidate during which OA disease stages, and under which conditions, longitudinal T2 changes occur in femorotibial cartilage, and whether some currently unknown phenomena may be in place that inhibit normal age-related increase of cartilage T2^2^,^33^ to be detectable in specific stages of early OA.

Radiography is frequently used for the enrollment of participant in studies, because radiographic scores (e.g. KLG or JSN) have been shown to discriminate between knees with and without subsequent cartilage loss^36^,^37^ and because the technique is affordable for large studies. Although systematically comparing cartilage T2 relaxation parameters between well-defined radiographic strata represents only one of several approaches necessary for qualifying cartilage T2 as a biomarker in (early) knee OA, we feel it is important to relate cartilage T2 to a well-accepted standard of structural staging of knee OA.

## Conclusion

In conclusion, this study did not identify differences in superficial or deep femorotibial cartilage T2 between knees with definite early ROA and contralateral knees without signs of ROA but with risk factors for developing OA. However, significant differences in T2 were detected between knees from subjects without vs. those with risk factors of OA. These differences were stronger for superficial than for deep cartilage T2 and prevailed when adjusting for age, sex and BMI. Over 1 year, a longitudinal increase in T2 was noted in the superficial and deep layers of healthy reference subjects without risk factors, but not in knees with early ROA or at risk of developing OA. These results suggest that differences in cartilage T2 previously observed between ROA and non-ROA cartilage may actually be due to differences in risk factor profiles between cohorts rather than to actual differences in ROA status.

## Additional Information

**How to cite this article**: Wirth, W. *et al*. Layer-specific femorotibial cartilage T2 relaxation time in knees with and without early knee osteoarthritis: Data from the Osteoarthritis Initiative (OAI). *Sci. Rep*. **6**, 34202; doi: 10.1038/srep34202 (2016).

## Figures and Tables

**Table 1:** Participant demographics.

		Risk ROA BL (n = 28)	(BL & Y1, n = 26)	Early ROA BL (n = 32)	(BL & Y1, n = 24)	Healthy BL & Y1 (n = 89)
Male	N/%	14/50.0%	(14/53.8%)	14/43.8%	(10/41.7%)	36/40.4%
Female	N/%	14/50.0%	(12/46.2%)	18/56.3%	(14/58.3%)	53/59.6%
No pain	N/%	14/50.0%	(12/46.2%)	10/31.3%	(8/33.3%)	89/100.0%
Infrequent pain	N/%	8/28.6%	(8/30.8%)	12/37.5%	(9/37.5%)	0/0.0%
Frequent pain	N/%	6/21.4%	(6/23.1%)	10/31.1%	(7/29.2%)	0/0.0%
Age [years]	Mean ± SD	61.1 ± 9.4	(60.7 ± 9.6)	60.2 ± 10.0	(61.5 ± 9.8)	55.0 ± 7.5
BMI [kg/m^2^]	Mean ± SD	28.0 ± 5.0	(28.1 ± 5.2)	27.6 ± 4.6	(27.4 ± 4.2)	24.4 ± 3.1
Weight [kg]	Mean ± SD	80.2 ± 15.6	(81.3 ± 15.5)	76.4 ± 15.5	(76.3 ± 13.6)	69.1 ± 12.0
Height [cm]	Mean ± SD	168.3 ± 8.4	(169.2 ± 7.9)	166.2 ± 9.2	(166.9 ± 9.3)	167.8 ± 8.8
minJSW [mm]	Mean ± SD	4.9 ± 1.0	(4.9 ± 1.0)	4.6 ± 0.8	(4.7 ± 0.7)	4.8 ± 0.8
MFTC.ThC [mm]	Mean ± SD	3.5 ± 0.5	(3.5 ± 0.5)	3.5 ± 0.5	(3.6 ± 0.5)	3.4 ± 0.5
LFTC.ThC [mm]	Mean ± SD	3.9 ± 0.5	(4.0 ± 0.4)	4.1 ± 0.5	(4.1 ± 0.5)	3.8 ± 0.5

BL = Baseline; Y1 = year 1 follow-up; SD = standard deviation; Risk ROA: Participants without ROA but with risk factors for ROA in the analyzed knee; Early ROA: Participants with early ROA in the analyzed knee; Healthy: Participants from the healthy reference cohort; Values in brackets show the demographic data for the participants from the risk ROA and early ROA group, for which no Y1 follow-up MR images were available. minJSW: Minimum radiographic joint space width from fixed-flexion x-rays. MFTC/LFTC.ThC: Mean cartilage thickness in the medial/lateral femorotibial compartment. The height measurement was missing for one of the participants from the risk ROA group.

**Table 2:** Baseline T2 values in knees without ROA but with risk factors for ROA (risk ROA), in knees with early ROA, and in healthy reference knees.

		Risk ROA (N = 28) Mean ± SD (95% CI)	Early ROA (N = 32) Mean ± SD (95% CI)	Healthy (N = 89) Mean ± SD (95% CI)	Risk ROA vs. Early ROA Crude/Adjusted Cohen’s D	Risk ROA vs. Healthy Crude/Adjusted Cohen’s D	Early ROA vs. Healthy Crude/Adjusted Cohen’s D
Avg	Deep	37.3 ± 1.8	37.3 ± 2.5	35.8 ± 1.8	0.983/0.984	<0.001/0.006	<0.001/0.011
		(36.6, 38.0)	(36.4, 38.2)	(35.4, 36.2)	0.01	0.81	0.73
	Superficial	48.1 ± 3.1	48.0 ± 3.5	45.4 ± 2.3	0.932/0.931	<0.001/0.001	<0.001/<0.001
		(46.9, 49.3)	(46.7, 49.3)	(44.9, 45.9)	0.02	1.04	0.96
MT	Deep	34.0 ± 1.8	33.3 ± 1.8	33.0 ± 1.9	0.140/0.137	0.020/0.401	0.511/0.509
		(33.3, 34.7)	(32.6, 33.9)	(32.6, 33.4)	0.39	0.51	0.14
	Superficial	43.6 ± 2.5	43.4 ± 2.9	41.6 ± 2.9	0.812/0.902	0.001/0.006	0.002/0.004
		(42.6, 44.5)	(42.4, 44.5)	(40.9, 42.2)	0.06	0.72	0.64
cMF	Deep	41.8 ± 3.8	42.5 ± 5.9	39.3 ± 3.4	0.595/0.628	0.001/0.008	<0.001/0.007
		(40.4, 43.3)	(40.4, 44.6)	(38.6, 40.1)	0.14	0.71	0.76
	Superficial	52.8 ± 5.8	53.4 ± 6.0	49.6 ± 3.8	0.662/0.683	0.001/0.027	<0.001/0.001
		(50.5, 55.0)	(51.3, 55.6)	(48.8, 50.4)	0.11	0.72	0.86
LT	Deep	32.0 ± 1.4	32.5 ± 2.1	31.0 ± 1.9	0.350/0.377	0.012/0.031	0.001/0.003
		(31.5, 32.6)	(31.7, 33.2)	(30.6, 31.4)	0.24	0.55	0.73
	Superficial	45.5 ± 3.6	44.5 ± 4.3	42.4 ± 2.6	0.368/0.390	<0.001/<0.001	0.001/0.006
		(44.1, 46.9)	(43.0, 46.1)	(41.9, 43.0)	0.23	1.07	0.67
cLF	Deep	41.2 ± 2.8	40.8 ± 3.2	39.7 ± 2.8	0.622/0.542	0.013/0.082	0.059/0.123
		(40.1, 42.3)	(39.7, 41.9)	(39.1, 40.3)	0.13	0.54	0.39
	Superficial	50.5 ± 4.1	50.7 ± 4.0	48.1 ± 2.9	0.901/0.969	0.001/0.007	<0.001/0.001
		(48.9, 52.1)	(49.2, 52.1)	(47.4, 48.7)	0.03	0.76	0.80

SD = standard deviation; Avg = average values across all four femorotibial cartilage plates; MT = medial tibia; cMF = weight-bearing (central) medial femur; LT = lateral tibia; cLF = weight-bearing (central) lateral femur; for a definition of the regions of interest, please also see Fig. 1.

**Table 3:** Longitudinal (one year) change in T2 values in knees without ROA but with risk factors for ROA (risk ROA), in knees with early ROA, and in healthy reference knees.

		Risk ROA (N = 26) Mean ± SD (95% CI)	Early ROA (N = 24) Mean ± SD (95% CI)	Healthy (N = 89) Mean ± SD (95% CI)	Risk ROA vs. Early ROA Crude/Adjusted Cohen’s D	Risk ROA vs. Healthy Crude/Adjusted Cohen’s D	Early ROA vs. Healthy Crude/Adjusted Cohen’s D
Avg	Deep	0.0 ± 1.7	−0.2 ± 1.1	**0.8 ± 1.3**	0.684/0.583	0.010/0.032	0.001/0.002
		(−0.7, 0.7)	(−0.6, 0.3)	**(0.5, 1.1)**	0.12	0.58	0.78
	Superficial	−0.4 ± 1.8	−0.1 ± 1.5	**0.5 ± 1.4**	0.581/0.694	0.006/0.024	0.041/0.029
		(−1.1, 0.3)	(−0.8, 0.5)	**(0.2, 0.9)**	0.16	0.62	0.48
MT	Deep	−0.3 ± 1.8	0.0 ± 1.6	**0.8 ± 1.7**	0.504/0.533	0.003/0.013	0.033/0.082
		(−1.1, 0.4)	(−0.7, 0.7)	**(0.5, 1.2)**	0.19	0.67	0.50
	Superficial	−0.4 ± 1.8	0.1 ± 1.8	**0.7 ± 1.8**	0.323/0.358	0.008/0.044	0.175/0.202
		(−1.2, 0.3)	(−0.7, 0.9)	**(0.3, 1.0)**	0.28	0.60	0.31
cMF	Deep	0.3 ± 2.9	−0.3 ± 2.5	**1.0 ± 2.1**	0.461/0.409	0.169/0.314	0.012/0.019
		(−0.9, 1.4)	(−1.3, 0.7)	**(0.5, 1.4)**	0.21	0.31	0.59
	Superficial	−0.5 ± 4.5	−0.5 ± 4.2	0.4 ± 2.6	0.993/0.904	0.195/0.219	0.196/0.060
		(−2.3, 1.3)	(−2.3, 1.3)	(−0.1, 0.9)	0.00	0.29	0.30
LT	Deep	−0.1 ± 1.9	−0.2 ± 0.9	**0.9 ± 1.5**	0.730/0.512	0.010/0.026	0.001/0.002
		(−0.9, 0.7)	(−0.6, 0.1)	**(0.5, 1.2)**	0.10	0.58	0.77
	Superficial	−0.2 ± 1.8	−0.1 ± 0.9	**0.6 ± 1.5**	0.872/0.937	0.024/0.089	0.025/0.041
		(−0.9, 0.5)	(−0.5, 0.3)	**(0.3, 0.9)**	0.05	0.51	0.52
cLF	Deep	0.1 ± 1.8	−0.2 ± 1.3	**0.6 ± 1.8**	0.545/0.516	0.182/0.227	0.038/0.023
		(−0.6, 0.9)	(−0.7, 0.4)	**(0.3, 1.0)**	0.17	0.30	0.48
	Superficial	*−0.5 ± 1.0*	0.0 ± 1.1	*0.6 ± 2.1*	0.133/0.138	0.020/0.056	0.212/0.334
		*(−0.9, −0.1)*	(−0.5, 0.4)	*(0.1, 1.0)*	0.43	0.52	0.29

SD = standard deviation; Avg = average values across all four femorotibial cartilage plates; MT = medial tibia; cMF = weight-bearing (central) medial femur; LT = lateral tibia; cLF = weight-bearing (central) lateral femur; for a definition of the regions of interest, please also see Fig. 1; significant change between baseline and one year follow up with p < 0.05 is marked in italics, and with p < 0.01 in bold letters.
